# An unusual presentation of multiple cavitated lung metastases from colon carcinoma

**DOI:** 10.1186/1471-2342-11-13

**Published:** 2011-05-31

**Authors:** Patrizia Seminara, Gaia Manna, Alessandra Emiliani, Alessandro Iannace, Tania Losanno

**Affiliations:** 1 Department of Internal Medicine, Oncology A Unit - University "Sapienza" Viale dell'Università 37 - 00185 Rome, Italy

**Keywords:** Excavated lung metastases, Computed tomography scan, Colon carcinoma, Atypical radiologic features of lung lesions, Diagnostic imaging

## Abstract

**Background:**

Consolidation with or without ground-glass opacity is the typical radiologic finding of lung metastases of adenocarcinoma from the gastrointestinal tract. Lung excavated metastases from gastrointestinal carcinoma are very rare.

**Case presentation:**

The authors describe an unusual presentation of multiple cavitated lung metastases from colon adenocarcinoma and discuss the outcome of a patient. The absence both of symptoms and other disease localizations, the investigations related to different diagnostic hypotheses and the empirical treatments caused a delay in correct diagnosis. Only a transparietal biopsy revealed the neoplastic origin of nodules.

**Conclusions:**

This report demonstrates that although lung excavated metastases are described in literature, initial failure to reach a diagnosis is common. We would like to alert clinicians and radiologists to the possibility of unusual atypical features of pulmonary metastases from colon adenocarcinoma.

## Background

Typical radiologic findings of pulmonary metastases include multiple round variable-sized nodules, generally located in peripheral parenchyma and diffuse thickening of interstitial [1,2]. Among cases of multiple nodules detected with CT-scan, 73% were reported to be pulmonary metastases [3]. The characteristic radiological findings of primary tumors and differential diagnoses of atypical lung metastases are reported by J. B. Seo et al. [4].

Consolidation with or without ground-glass opacity is the typical radiologic finding of lung metastases of adenocarcinoma from the gastrointestinal tract. Cavitating lesions are detected only in 4% of metastatic nodules and about 70% of them are due to metastatic squamous cell carcinoma. Lung excavated metastases from osteosarcoma are very rare and those from adenocarcinomas of various primary sites (gastrointestinal, breast, ovary, etc) are only occasional.

Herein the authors report a case of excavated pulmonary metastases from colon adenocarcinoma, whose unusual presentation and the absence both of symptoms and other sites of disease caused delay of correct diagnosis.

## Case presentation

C.E., a 78 year-old Caucasian woman, underwent radical surgery for adenocarcinoma of the sigma (pT4, pN1, and G2). The patient presented a history of coronary heart disease, but at that time she had been taking a long action nitrate, she was free of symptoms even when exposed to moderate physical exercises and ECG was normal. She completed six cycles of adjuvant chemotherapy with Raltitrexed (3 mg/sqm every 21 days), a good tolerable treatment.

**Figure 1:**
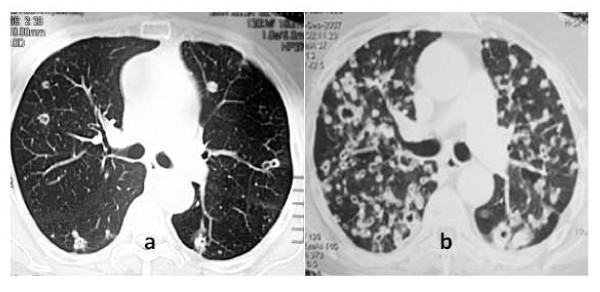
**Atypical radiologic findings of excavated pulmonary metastases: initial lung CT scan (a) compared with the picture (b) after 7 months of unsuccessful chemotherapy**.

**Figure 2:**
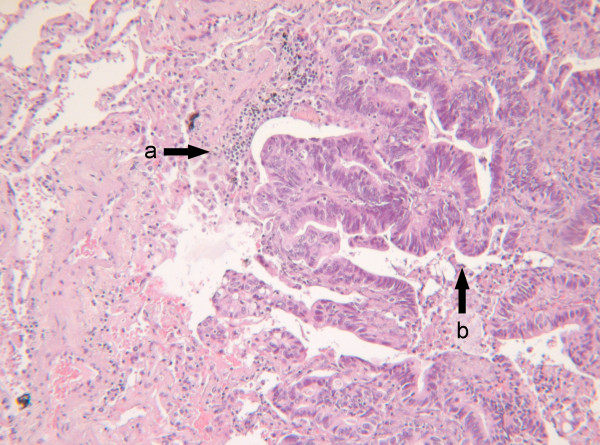
**Histological findings of percutaneous transthoracic biopsy showing metastatic cells infiltrating into lung parenchyma (a) and recognizable glandular structures of tumor cells from colon adenocarcinoma (b)**. Original magnification, x100; hematoxylin-eosin stain.

Follow up controls were negative for two years, when a planned chest radiograph revealed the presence of patchy opacities. The patient was asymptomatic and clinical examination was unremarkable. Tumor serum markers and inflammatory indices (erythrocytes sedimentation rate, C-reactive protein) were normal. White blood cells count (9,680 cells/mcL) was within the normal reference range. A CT-scan (Figure 1a) confirmed the presence of multiple lung lesions mainly measuring less than 1cm with colliquative central necrosis. Infectious disease consultants suspected an invasive aspergillosis and the serum galactomannan assay was required. This test was negative as well as the neoplastic cell detection in B.A.L. A staphylococcal pneumonia or a nocardiosis were proposed as possible alternative diagnosis and an empirical therapy with trymethoprim-sulphametoxazole was started. Finally, only a percutaneous transthoracic needle aspiration biopsy of the unmodified nodules allowed the diagnosis of EGFR positive colon carcinoma metastases (Figure 2). Therefore the patient was treated with sequential regimens of chemotherapy, five courses of Irinotecan-Bevacizumab regimen and eight courses of Oxaliplatin-Cetuximab regimen. No response was observed and the last CT-scan (Figure 1b) depicted a miliariform diffusion of the process to both lungs with further enlarged excavated lesions. The patient died after one month.

## Conclusions

The lung is a common site for metastases. Most cases of pulmonary metastases can be diagnosed radiologically on the basis of typical findings, including multiple round variable-sized nodules and a diffuse thickening of interstitium, respectively hematogenous metastases and lymphangitic carcinomatosis. In case of atypical radiologic features of pulmonary metastases, it is crucial to carry out an accurate diagnosis. Differential diagnostic hypotheses for these appearances include infective conditions either bacterial or fungal in origin, rheumatoid nodules and vasculitic processes, lymphomas and all metastatic diseases from a distal primary tumor [4,5].

As regards adjuvant chemotherapy with Raltitrexed, our patient was elderly with a previous personal history of coronary disease. This therapeutic choice was in relation to the results of three randomized trials [6-8], demonstrating equivalence in response rate of Raltitrexed and 5-FU/leucovorin schedules in patients with advanced colorectal carcinoma. There is a small statistically significant difference for time to progression and, in one study a shorter median survival, but these minimal differences may not be clinically relevant for an individual therapeutic decision, considering also that patients with coronary disease are more likely to experience 5-FU cardiotoxicity [9].

In our case, first diagnostic hypothesis was pulmonary infection, featuring very limited lung excavated lesions at a follow-up planned chest radiograph. The absence both of symptoms and other disease localizations by CT-scan contributed to infectious diagnosis. But investigations and empiric treatment caused a delay of correct diagnosis and treatment. Therefore, only biopsy of lung lesion allowed the diagnosis of EGFR positive colon carcinoma metastases (Figure 2). The mis-diagnosis of infection diseases has been reported in literature for 10% of metastatic lung adenocarcinomas presenting the features of multiple cavitating nodules [10].

Considering the biology of cancer this case history shows its polyphenotipic nature. Despite the grading (G2), this phenotype of cancer appeared to be rather aggressive, characterized by devastating course, absence of serum tumor markers increase and resistance to chemotherapy based on updated agents [11]. Progress in molecular biology and its use in clinical practice are strongly justified to identify patients with peculiar clinical course and prognosis.

According to our experience and literature data, in daily practice unusual cavitated lung metastases are not rare and it is often difficult to distinguish metastases from other non-malignant pulmonary diseases. In this case only multiple excavated lung nodules were found, without other sites of metastasis, and the mis-diagnosis of infectious disease caused a delay in administering effective anticancer therapy. After histological diagnosis of lung metastases from primary colon adenocarcinoma, the first CT-scan was submitted again to other radiologists who confirmed the difficulty in interpreting initial features of cavitated lung lesions.

In conclusion, although other cases of cavitated lung metastases are reported in literature, these atypical secondary lung lesions are still diagnosed late. The aim of this report is to alert clinicians and radiologists to the possibility of unusual features of pulmonary metastases due to colon adenocarcinoma.

## Consent

Written informed consent was obtained from her relatives for publication of this case report and any accompanying images.

## Competing interests

The authors declare that they have no competing interests.

## Authors' contributions

PS, TL and AE were in charge of overall care of the patient and drafted the manuscript. GM and AI participated in literature review, collected TC-scans and photomicrograph of lung biopsy. All authors revisited the manuscript critically for important intellectual content, read and approved the final manuscript.

## Pre-publication history

The pre-publication history for this paper can be accessed here:

http://www.biomedcentral.com/1471-2342/11/13/prepub
